# RHO OF PLANTS signalling and the activating ROP GUANINE NUCLEOTIDE EXCHANGE FACTORS: specificity in cellular signal transduction in plants

**DOI:** 10.1093/jxb/erae196

**Published:** 2024-04-29

**Authors:** Philipp Denninger

**Affiliations:** Plant Systems Biology, School of Life Sciences, Technical University of Munich, Emil-Ramann-Strasse 8, 85354 Freising, Germany; Academy of Sciences of Czech Republic, Czech Republic

**Keywords:** Auxin, cell division, cell polarity, membrane domain, phytohormones, RECEPTOR LIKE KINASEs (RLKs), ROP, ROPGEF, signalling, tip-growth

## Abstract

Every cell constantly receives signals from its neighbours or the environment. In plants, most signals are perceived by RECEPTOR-LIKE KINASEs (RLKs) and then transmitted into the cell. The molecular switches RHO OF PLANTS (ROP) are critical proteins for polar signal transduction and regulate multiple cell polarity processes downstream of RLKs. Many ROP-regulating proteins and scaffold proteins of the ROP complex are known. However, the spatiotemporal ROP signalling complex composition is not yet understood. Moreover, how specificity is achieved in different ROP signalling pathways within one cell still needs to be determined. This review gives an overview of recent advances in ROP signalling and how specificity by downstream scaffold proteins can be achieved. The composition of the ROP signalling complexes is discussed, focusing on the possibility of the simultaneous presence of ROP activators and inactivators within the same complex to balance ROP activity. Furthermore, this review highlights the function of plant-specific ROP GUANINE NUCLEOTIDE EXCHANGE FACTORS polarizing ROP signalling and defining the specificity of the initiated ROP signalling pathway.

## Teaser

This review summarizes recent advances in RHO OF PLANTS signalling, highlighting that distinct cellular functions depend on signalling complex composition and that ROP GUANINE NUCLEOTIDE EXCHANGE FACTORS determine signalling specificity.

## Introduction

Every living cell constantly receives different signals from its environment. Especially in multicellular organisms, cells must recognize and process many signals that influence their development, differentiation, and activity in response to nutrient availability or environmental stresses. To perceive such signals, each cell needs a specific set of signalling components for every trigger. Generally, every cellular signalling pathway consists of a receptor that recognizes the signal, components that transduce the perceived signal to the corresponding location in the cells, and effectors that react and change according to the perceived signal. However, a cell perceives multiple signals simultaneously, and most signalling pathways are not uncoupled from one another. Some signals can be perceived by various receptors, and individual receptors recognize different signals. Moreover, each signal can trigger multiple effectors using multiple transduction components, which overlap between individual signalling pathways (Liang and Zhou, 2018).  To comprehend an organism and its complex behaviour, we must fully understand the individual signalling components and characterize their specific functions and differences. In eukaryotic cells, Rho GTPases are molecular switches with a key function in signal transduction (Etienne-Manneville, 2004). Rho GTPase pathways are essential for many directional processes like cellular growth or immune responses. Therefore, Rho GTPase-dependent signalling is a primary model for understanding polar signal transduction and cellular signalling (Feiguelman *et al.*, 2018; Nielsen, 2020).

This review summarizes Rho GTPase signalling in plants and its critical functions for cell polarity and polar signalling. It discusses the formation, composition and structure of the RHO OF PLANTS (ROP) signalling complex. Moreover, it focuses on Rho GTPase-activating GUANINE NUCLEOTIDE EXCHANGE FACTOR (GEF) proteins, examining their function as the main components that drive the polarity of this signalling pathway and their potential to bring specificity into the complexity of cell signal transduction.

## Signal perception in plants

In plant cells, many external signals are perceived by plasma membrane-localized receptor proteins. Compared with other eukaryotes, plants have highly diversified the RECEPTOR-LIKE KINASE (RLK) proteins, which consist of an extracellular receptor domain, a single transmembrane domain and an intracellular kinase domain. The different classes and families of these RLKs are defined by their extracellular domains, and an overview of them is given by Dievart *et al.* (2020). The intracellular domain mainly consists of a kinase domain, which phosphorylates other proteins and, thus, can quickly and reversibly modify downstream proteins and initiate signal transduction. Some RLKs contain a pseudokinase domain that is not enzymatically active and cannot activate downstream signalling by phosphorylation. It remains to be clarified if all signalling pathways require kinase activity. Still, if the RLKs do not have kinase activity, they often recruit other RLKs as co-receptors, RLK-like proteins with active kinase domains, or cytosolic membrane-associated protein kinases (Liang and Zhou, 2018; Dievart *et al.*, 2020).  This demonstrates the importance of kinase activity for most known signalling pathways. After signal perception and the formation of an active receptor complex, the signal is transduced to downstream signalling components, which, in turn, trigger the release of second messengers like Ca^2+^ or alter the activity of proteins like transcription factors to induce specific cellular responses (Denninger *et al.*, 2014; Tang *et al.*, 2017; Liang and Zhou, 2018; Brost *et al.*, 2019). Typical downstream pathways are kinase cascades like the MITOGEN-ACTIVATED PROTEIN (MAP) KINASE pathway or pathways of small molecular switches like the ROP pathways. These signal transduction pathways usually comprise multiple homologs within one cell (Cristina *et al.*, 2010; Nielsen, 2020). Additionally, some RLK complexes are known to interact with proteins of different signalling pathways, and this is likely the case for most RLK-dependent pathways (Tang *et al.*, 2017).  The persistence of the interaction with the downstream signalling components and the specificity between the individual homologs and the different pathways still need to be studied.

## Rho GTPase signalling in plants

**Fig. 1.**
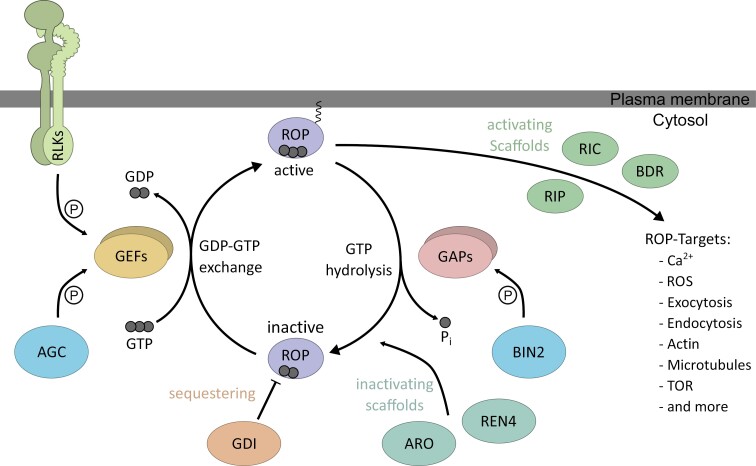
General model of the RHO OF PLANTS (ROP) signalling pathway. GUANINE NUCLEOTIDE EXCHANGE FACTORS (GEFs) activate ROPs by catalysing the exchange of GDP for GTP, which enhances ROP plasma membrane attachment. In plants, three classes of GEFs, which have characteristic catalytic GEF domains, can activate ROPs. SPIKE contains a DOCK domain, SWITCHING B-CELL COMPLEX-ASSOCIATED PROTEIN 70 (SWAP70) contains a DBL domain, and ROPGEFs contain the plant-specific PRONE domain. Active ROPs recruit different scaffolding proteins of the ROP INTERACTING CRIB-CONTAINING PROTEIN (RIC), ROP INTERACTING PARTNER (RIP), or BOUNDARY OF ROP DOMAIN (BDR) families to activate downstream signalling pathways. Depending on the complex composition and the recruited scaffold, specific pathways can be activated. GTPase ACTIVATING PROTEINS (GAPs) inactivate ROPs by promoting the hydrolysis reaction of ROPs from GTP to GDP. In plants, two classes of GAPs are found: PH domain-containing PHGAPs and plant-specific ROPGAPs, of which most contain a CRIB domain. Interaction of GAPs with ROPs can be enhanced by ARMADILLO REPEAT ONLY (ARO) or ROP1 enhancer 4 (REN4) scaffolding proteins. Furthermore, GUANOSINE NUCLEOTIDE DISSOCIATION INHIBITORs (GDIs) can sequester ROPs in the cytosol to ensure their inactivity. Both the activation and inactivation can be regulated by phosphorylation of the ROP regulators. GEFs are phosphorylated and activated by RECEPTOR-LIKE KINASEs (RLKs) and ACGVIII kinases, while GAPs are stabilized by phosphorylation via BRASSINOSTEROID-INSENSITIVE2 (BIN2). ROS, reactive oxygen species; TOR, TARGET OF RAPAMYCIN.

Rho GTPases are small molecular switches that act as signal transduction proteins and are conserved throughout eukaryotes. Plants gained a specific family of Rho GTPases, defined by the presence of a SYRGA motif and an evolved 12-amino-acid-long Rho insert region, which is found already in streptophyte algae and is present in all land plants. In most flowering plant species, this ROP family is highly diversified; for example, *Arabidopsis thaliana* has 11 ROPs (Berken, 2006; Mulvey and Dolan, 2023a). Unless otherwise noted, the proteins discussed in the review (e.g. ROP2, ROPGEF3) are from *Arabidopsis thaliana*, the experimental model in which ROP signalling has been most extensively characterized. The biochemical and cell biological function of ROPs are comparable to those of other Rho GTPases (Fig. 1). They bind guanosine triphosphate (GTP) and hydrolyse it to guanosine diphosphate (GDP), but their intrinsic enzymatic activity is very low. GTPase ACTIVATING PROTEINs (GAPs) promote the hydrolysis reaction of Rho GTPases from GTP to GDP, while GUANINE NUCLEOTIDE EXCHANGE FACTORS (GEFs) catalyse the exchange of GDP for GTP. ROPs change their cell biological activity state depending on the bound nucleotide and can activate downstream pathways. ROPs are considered cell biologically inactive when GDP is bound and active when GTP is bound. Thus, GAP proteins inactivate, and GEF proteins activate ROP signalling (Berken, 2006; Feiguelman *et al.*, 2018). In turn, the activity of both regulators is controlled by post-translational modifications, and to date GAP and GEF proteins have been found to be phosphorylated. GEFs are phosphorylated by RLKs and cytosolic AGCVIII protein kinases. The phosphorylation by RLKs at their C-terminus activates GEFs, while the effect of phosphorylation by AGC kinases is not understood (Kaothien *et al.*, 2005; E. Li *et al*., 2018). GAPs were shown to be phosphorylated by the cytosolic kinase BRASSINOSTEROID-INSENSITIVE2 (BIN2), a negative regulator of brassinosteroid signalling. This phosphorylation enhances GAP protein stability and localization, leading to more robust ROP inactivation (Zhang *et al*., 2022). Along with the change in activity, ROPs also change their localization within the cell (Fig. 1). While inactive ROPs are considered more cytosolic and sequestered by GUANOSINE NUCLEOTIDE DISSOCIATION INHIBITOR (GDI) proteins, active ROPs are more plasma membrane-associated (Bloch and Yalovsky, 2013; Feiguelman *et al.*, 2018).  This plasma membrane attachment is facilitated by a positively charged K/R-rich C-terminus and lipid modifications. Type I ROPs are defined by a C-terminal CaaX motive, which is prenylated at the ER membrane by geranylgeranyl transferases that attach a geranyl-geranyl lipid moiety at the cysteine. Type II ROPS do not contain such a prenylation site but a GC-CG box at their C-terminus in which two cysteines can be *S*-acylated to facilitate membrane attachment (Yalovsky, 2015). All ROPs (type I and type II) can also be palmitoylated on cysteines at the Golgi membrane. This modification is reversible and likely is a regulatory mechanism for membrane attachment and localization of ROPs into membrane domains. The sorting of ROPs into specific membrane domains and a resulting change in protein mobility is also driven by the association of the K/R-rich C-terminus with negatively charged phospholipids in the plasma membrane (Berken and Wittinghofer, 2008; Yalovsky, 2015; Feiguelman *et al.*, 2018; Noack and Jaillais, 2020). This nanodomain association is crucial for ROP6 in Arabidopsis and might be one mechanism to bring specificity into different ROP signalling pathways in the same cell and activate specific downstream targets (Platre *et al.*, 2019).

## Downstream specificity of ROP signalling

**Fig. 2.**
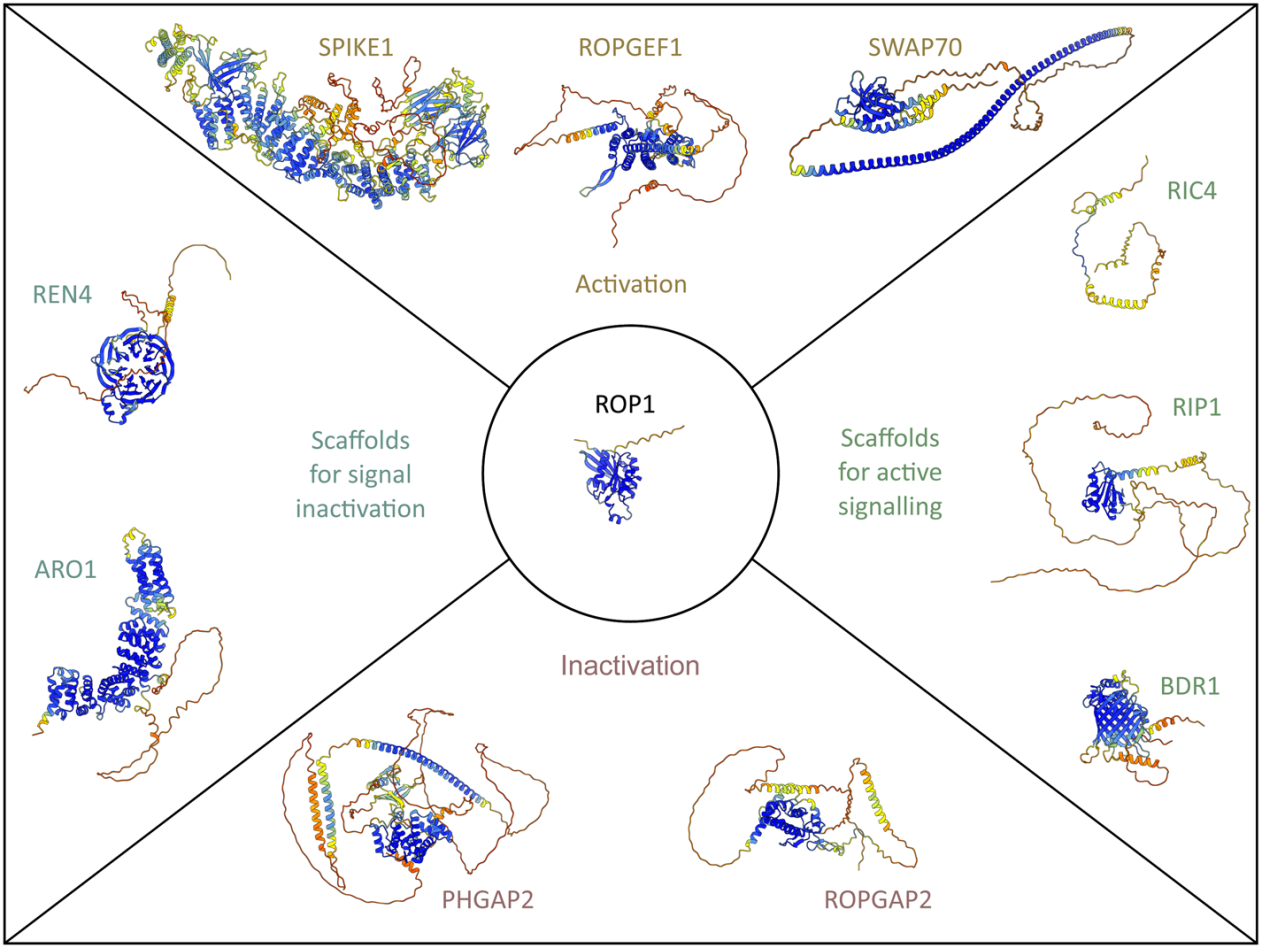
Protein structure predictions of RHO OF PLANTS (ROP) signalling components that form a complex with ROPs. Protein structures predicted by AlphaFold for one representative of each class of ROP regulators that directly interacts with ROPs and forms a signalling complex. For size comparison, all proteins are shown to the same scale. Likely, activating GUANINE NUCLEOTIDE EXCHANGE FACTORS (GEFs) and activating scaffold proteins co-occur within the same complex, together with inactivating GTPase ACTIVATING PROTEINs (GAPs) and scaffold proteins. Structures were obtained from https://alphafold.ebi.ac.uk/, and the most likely structure model is shown (Jumper *et al.*, 2021; Varadi *et al.*, 2022). Colour coding represents the pLDDT value of the model structure confidence, with blue representing high confidence and orange representing low confidence. Predicted structures of multi-domain proteins [e.g. ROPGAPs, PLECKSTRIN HOMOLOGY GTPase-ACTIVATING PROTEINs (PHGAPs), SWITCHING B-CELL COMPLEX-ASSOCIATED PROTEIN 70 (SWAP70)] with flexible sequences linking the individual domains need to be taken with caution. The prediction of the individual domains might be correct, but the orientation towards each other is very uncertain. The predicted aligned error (PAE) data should be considered to assess the inter-domain accuracy of the predicted structures. All shown proteins are from *Arabidopsis thaliana* with the following gene IDs and AlphaFold/Uniport model ID: ROP1 (AT3G51300, P92978), SPIKE1 (AT4G16340, Q8SAB7), ROPGEF1 (AT4G38430, A0A2H9ZS80), SWAP70 (AT2G30880, Q8VYJ6), RIC4 (AT5G16490, Q9FFD5), RIP1 (At1G17140, A0A178VHP9), BDR1 (AT5G06610, Q9FG09), ROPGAP2 (AT4G03100, F4JI46), PHGAP2 (AT5G19390, F4K142), ARO1 (AT4G34940, Q9SW41), REN4 (AT2G26490, O48716).

ROPs serve as molecular switches, transducing signals perceived by RLKs to effector proteins. Different downstream effectors and associations to other signalling pathways and cellular functions have been described. ROP signalling amongst other things is in a feedback loop with Ca^2+^ signalling in tip-growing cells; activates reactive oxygen species (ROS) production by NADPH OXIDASES (NOXs)/RESPIRATORY OXIDASE HOMOLOGS (RBOHs) in multiple cell types and processes; is in a feedback loop with phospholipid signalling; positively or negatively regulates actin and microtubule polymerization depending on the ROP and cell type; transduces brassinosteroid signals in pavement cells; is in a negative feedback loop with abscisic acid signalling; inhibits endocytosis of PINFORMED auxin transporters; generally promotes exocytosis via the exocyst complex; and transduces TARGET OF RAPAMYCIN (TOR) metabolic signalling (Fu *et al.*, 2001; Hwang *et al.*, 2005; Jones *et al*., 2007; Lavy *et al.*, 2007; Kusano *et al.*, 2008; Wong *et al.*, 2008; Hazak *et al.*, 2010; Xu *et al.*, 2010; Li *et al*., 2012; Potocký *et al.*, 2012; Schepetilnikov *et al.*, 2017; Brost *et al.*, 2019; Lauster *et al.*, 2022; Zhang *et al*., 2022). A detailed description of many of these downstream effectors is provided by Feiguelman *et al.* (2018), but exceeds the scope of this review. Here, I want to emphasize that only a few ROP proteins are expressed in one cell and they are mainly considered redundant (Xiang *et al.*, 2023). Still, these ROPs simultaneously regulate multiple pathways within one cell and specifically address individual cellular functions (Gu *et al.*, 2005). The specific regulation of downstream pathways by ROPs is mediated by scaffolding proteins, which can be divided into three major groups (Figs 1, 2). ROP INTERACTING CRIB-CONTAINING PROTEINS (RICs) are defined by a CDC42/RAC INTERACTIVE BINDING (CRIB) motif, which is a conserved feature in different eukaryotic proteins and is required for Rho GTPase binding specifically in the active form (Wu *et al.*, 2001). Besides this, RICs are generally small and unstructured (Fig. 2). The family of ROPGAPs in plants also contains a CRIB motif, giving them stronger ROP binding capacities than the other major family of ROP-interacting GAPs in plants, the PLECKSTRIN HOMOLOGY GTPase-ACTIVATING PROTEINs (PHGAPs) (Wu *et al.*, 2000; Ruan *et al.*, 2023). While ROPGAPs contain CRIB and GAP domains as conserved domains, the GAP domain of PHGAPs is flanked by an N-terminal pleckstrin homology (PH) domain, which facilitates membrane attachment, and a long flexible C-terminus containing coiled-coiled domains, which were shown to facilitate protein–protein interactions (Fig. 2) (Schaefer *et al.*, 2011; Stöckle *et al.*, 2016). It is important to note that the predicted structure of multi-domain proteins like ROPGAPs and PHGAPs with flexible regions between the individual domains must be taken cautiously. Even though the individual domains might be predicted correctly, their orientation towards one another is very uncertain, as seen in the confidence values of the structures shown in Fig. 2. The second group of scaffolding proteins is called MICROTUBULE DEPLETION DOMAIN (MIDD), INTERACTOR OF CONSTITUTIVELY ACTIVE ROP (ICR), or ROP INTERACTING PARTNER (RIP), and does not contain conserved sequence features but is rather characterized by coiled-coiled domains (Lavy *et al.*, 2007; Li *et al*., 2008; Oda and Fukuda, 2012). Compared to RICs, RIPs are more structured but still contain large, flexible regions (Fig. 2). The third group are BOUNDARY OF ROP DOMAIN (BDR) proteins, defined by a DUF620 domain of unknown function (Sugiyama *et al*., 2019). BDR proteins interact directly with ROPs, but their structure is much more defined and compact than RICs and RIPs (Fig. 2). These families of scaffolding proteins also diversified in flowering plants, with, for example, 11 RIC, five RIP, and nine BDR proteins in *Arabidopsis thaliana*, allowing the formation of different ROP complexes. Associating with the same ROP protein, different scaffolding proteins can activate specific pathways, as shown in tobacco pollen tubes, where RIC3 activates Ca^2+^ signalling and RIC4 activates actin accumulation (Gu *et al.*, 2005). Similar specificities towards effectors were also demonstrated for RIP proteins. All RIP proteins are associated with microtubules in Arabidopsis; however, only RIP1, RIP3, and RIP4, but not RIP2 and RIP5, are associated with the pre-prophase band during cell division (Hasi and Kakimoto, 2022). However, it still needs to be determined which molecular mechanisms in these complexes lead to the different interactions and activation of downstream pathways. This highlights that the goal of understanding ROP signalling, its specificity, and relevant downstream pathways, in general is to know the entire signalling complex, including all regulating proteins and scaffold proteins, in a spatiotemporally resolved manner.

## The complexity of the ROP signalling complex

The classical view of a signalling pathway like the ROP activity cycle is temporally linear. ROP proteins are activated by GEFs and interact with RICs and RIPs to activate downstream pathways. Afterwards, they are inactivated by GAPs, which causes RIPs and RICs to dissociate, and signal transduction is stopped (Fig. 1) (Berken, 2006; Feiguelman *et al.*, 2018). However, multiple studies show evidence for a more complex and less linear mechanism. Even though there are specificities for the interaction with one or the other activity status of ROPs, certain ROPGEFs and ROPGAPs can interact with both forms of ROPs, as was shown for ROPGEF3 and ROP2 interaction in Arabidopsis and ROPGAP1 and ROP4 of *Physcomitrium patens* (Denninger *et al.*, 2019; Ruan *et al.*, 2023). Similar interactions with both forms of ROPs were also demonstrated for the BDR scaffolding proteins (Xiang *et al.*, 2023). However, not all BDR proteins seem able to interact with active and inactive ROPs to a similar extent, as BDR1 only interacts with active ROPs. BDR8 and BRD9 interact with both forms, but BDR8 preferentially interacts with active ROPs, while BDR9 interacts with both the same (Sugiyama *et al*., 2019; Xiang *et al.*, 2023). A strong discrimination between the different forms of ROPs was also shown for the scaffolding proteins ARMADILLO REPEAT ONLY (ARO) and the WD40 domain containing JINGUBANG (JGB)/ROP1 enhancer 4 (REN4) (H. Li *et al.*, 2018; Kulich *et al.*, 2020). REN4 interacts with components of clathrin-mediated endocytosis during pollen tube growth and suppresses ROP activity. AROs interact with GAP proteins and confine ROP activity during root hair formation and likely other cells and processes. These scaffolding proteins partially co-localize with ROPs, and both were shown to have a strong affinity for interacting with the active form of ROPs in pull-down experiments (H. Li *et al.*, 2018; Kulich *et al.*, 2020). Despite the strong preference towards active ROPs, AROs and REN4 can, to a lesser extent, interact with inactive ROPs. This suggests that these scaffolding proteins can also remain in inactivated ROP complexes. This shows that the binding capacity of scaffolding proteins towards the different activity forms of ROPs can vary. We will need to analyse these differences in ROP binding quantitatively to understand ROP complex formation and the specification in signalling output. Direct ROP regulators and scaffold proteins of the ROP complex both have the potential to interact with active and inactive ROPs (Fig. 2). This suggests that ROP complexes do not undergo a full exchange of regulators but instead form a large complex of multiple regulators including activators and inactivators simultaneously. This has the advantage of fast switching of the ROP activity status, which limits the time of uncontrolled ROP activation and allows precise spatiotemporal regulation of ROP activity (Hwang *et al.*, 2005; Nagawa *et al.*, 2010; Qin and Yang, 2011; Portes *et al.*, 2015). In unstable systems like tip-growing root hairs and pollen tubes, this fine-tuning of the growth machinery is essential to maintain constant growth while maintaining cellular integrity (Nagawa *et al.*, 2010; Portes *et al.*, 2015). Thus, the ROP signalling complex is likely a permanent protein association in which the small molecular switch is constantly surrounded by larger regulators and scaffold proteins. The different activation states of the central ROP would lead to altered binding preferences of scaffolds and likely conformational changes favouring different downstream outputs. Such stable complexes with different compositions have the advantage of a more reliable and specific association with their downstream partners. ROP complexes with varying compositions of ROPGEFs and ROPGAPs can lead to different localizations and formation of specific membrane domains (Sternberg *et al.*, 2021), which increases the potential formation of ROP complexes with specific signal transduction properties. Different ROPGAPs and ROPGEFs have different binding capacities towards individual ROPs *in vitro*, which can indicate naturally occurring complex compositions (Berken *et al.*, 2005; Schaefer *et al.*, 2011). Still, there needs to be confirmation of these binding preferences *in vivo*. Understanding the composition and nature of different ROP complexes requires detailed simultaneous analysis of multiple complex components *in vivo* and as protein structures of the ROP signalling complex. Advances in protein–protein interaction studies by proximity labelling or protein complex structure analysis will help with these challenging tasks (Bagci *et al.*, 2020; Ganesan *et al.*, 2020; Zhang *et al*., 2020; Günsel and Hagn, 2022; Mirdita *et al.*, 2022). This will allow us to comprehend the complexity and specificity of ROP signalling and how this pathway can regulate the different spatiotemporal aspects of polar signalling.

## ROPGEFs are plant-specific activators of ROP signalling

Rho GTPases are conserved in all eukaryotes and require activation by GEFs. In animals and fungi, two main types of GEFs evolved and diversified. DIFFUSE B CELL LYMPHOMA (DBL)-type GEFs in which the DBL-HOMOLOGY (DH) domain confers GEF activity, or CED5/DOCK180 ZIZIMIN HOMOLOGY (CZH) RhoGEFs in which the DOCKER (DOCK) domain confers GEF activity (Meller *et al.*, 2005; Rossman *et al.*, 2005). GEF activity was shown for plant homologs of these GEFs, like the DOCK-GEF SPIKE1 of Arabidopsis and the rice DBL-GEF SWITCHING B-CELL COMPLEX-ASSOCIATED PROTEIN 70 (SWAP70) (Fig. 2; Qiu *et al.*, 2002; Basu *et al.*, 2008; Yamaguchi *et al.*, 2012). For these large proteins with multiple domains connected by flexible linkers, the domain arrangement seen in the structure prediction of Fig. 2 is very uncertain and needs to be taken with caution. However, plants usually contain only a few copies of these GEFs, and they did not diversify similarly to those of animals and fungi. Plants evolved the specific family of ROP proteins, which is functionally homologous to other Rho GTPases but contains small sequence specificities (Berken *et al.*, 2005; Gu *et al.*, 2006; Feiguelman *et al.*, 2018). The ROP family originated in the streptophyte lineage and diversified in land plants (Mulvey and Dolan, 2023a). Similarly, plants evolved a new family of GEF proteins characterized by a PLANT-SPECIFIC ROP NUCLEOTIDE EXCHANGER (PRONE) domain. These PRONE domain proteins also evolved in the streptophyte lineage. Still, the exact origin is unclear, as PRONE-like proteins with limited sequence similarity were also found in other Archaeplastida lineages (Elias, 2008; Mulvey and Dolan, 2023a). In land plants, these PRONE domain-containing proteins diversified much more than DBL or DOCK GEFs, with, for example, six PRONE GEFs in *Physcomitrium patens*, 10 in *Medicago truncatula*, 14 in *Arabidopsis thaliana*, and 11 in *Oryza sativa* (Berken *et al.*, 2005; Eklund *et al.*, 2010; Riely *et al.*, 2011; Kim *et al.*, 2020). The PRONE domain proteins were named ROPGEFs, as they have GEF activity specifically towards ROPs but not to other types of Rho GTPases (Berken *et al.*, 2005). As DBL GEFs show only weak GEF activity towards ROPs compared to ROPGEFs, it is possible that PRONE domain-containing proteins evolved along with the emergence of ROPs to maintain a high capacity for ROP activation. The PRONE domain structure of GEF8 was solved in complex with ROP4, which revealed the crucial residues for the catalytic function and that these GEFs occur as dimers, which is necessary for their functionality (Thomas *et al.*, 2007). The activity of the PRONE domain can be regulated by AGCVIII-dependent phosphorylation, but the exact molecular mechanism of this regulation is still not understood (Zhang *et al*., 2009; Li *et al*., 2018; Li *et al.*, 2020). Variable termini with no predicted structural features usually flank the conserved PRONE domain (Berken *et al.*, 2005; Thomas *et al.*, 2007). Structure predictions indicate that both termini are likely unstructured and flexible (Bouatta *et al.*, 2024, Preprint). It was suggested that these flexible terminal domains undergo conformational changes upon post-translational modifications or interactions with other proteins, as these termini have regulatory functions for the GEF activity (Zhang and McCormick, 2007; Chang *et al.*, 2013). While the N-terminus is thought to positively affect GEF activity, the C-terminus inhibits the activity of the PRONE domain. This inhibition can be released by phosphorylating the C-terminus and interaction with RLKs like POLLEN RECEPTOR LIKE KINASEs (PRKs) or the malectin-like domain-containing RLK FERONIA (FER) (Zhang and McCormick, 2007; Chang *et al.*, 2013; Zhao *et al.*, 2013; Tang *et al.*, 2022). Moreover, the C-terminus is responsible for the subcellular localization of some ROPGEFs, as shown for the polarization of ROP signalling during pollen germination (Bouatta *et al.*, 2024, Preprint). However, some ROPGEFs do not have this variable C-terminus. The Arabidopsis ROPGEF3 and ROPGEF4 polarize and activate ROPs during root hair growth, but both lack a variable C-terminal domain (Denninger *et al.*, 2019). How these proteins polarize and which mechanism activates their function still needs to be investigated.

## ROP signalling is polarized by ROPGEF accumulation

Signal transduction by Rho GTPases requires transmitting the perceived signal to these molecular switches. For this, Rho GTPases must be recruited to active receptors and be activated by GEF proteins. Thus, GEFs are crucial in this pathway as they initiate Rho GTPase activation after signal perception by receptors (Bos *et al.*, 2007). In plants, ROPs are involved in many cell polarity processes. ROP signalling was studied extensively, especially in highly polar tip-growing cells, and ROPGEF proteins were shown to activate ROPs in these systems (Lin *et al.*, 1996; Jones *et al.*, 2002; Li *et al*., 2002; Kaothien *et al.*, 2005; Denninger *et al.*, 2019; Bouatta *et al.*, 2024, Preprint). Additionally, the two tip-growing cell types, root hairs and pollen tubes, serve as models to study the *de novo* establishment of a subcellular polar growth domain. We showed recently that ROPGEFs are the earliest known factors that polarize at the root hair initiation site and initiate the recruitment of ROPs and subsequent growth regulators (Denninger *et al.*, 2019). Furthermore, ROPGEFs accumulate in germinating pollen grains minutes before germination, defining this site and activating ROP-dependent signalling pathways (Bouatta *et al.*, 2024, Preprint). This shows that ROPGEFs can polarize and recruit ROPs before growth is initiated. How this polarization is achieved and whether this mechanism is conserved or specific for each process still needs to be studied. During pollen germination, the C-terminal domain of ROPGEF8 and ROPGEF9 was responsible for this polarization, suggesting recruitment to RLKs (Bouatta *et al.*, 2024, Preprint). However, the polarization of ROPGEF3 in root hairs must be achieved by a different mechanism, as this ROPGEF does not have a variable C-terminal region. ROPGEF3 forms nanodomains of low mobility at the plasma membrane of root hair cells, which recruit ROP2 (Denninger *et al.*, 2019; Fuchs *et al.*, 2021, Preprint). However, it still needs to be determined if these nanodomains form by intrinsic properties of ROPGEF3 or their formation depends on additional factors, like scaffolding proteins or clustering of specific phospholipids as described for ROP6 containing nanodomains (Platre *et al.*, 2019).

## ROPGEFs define the specificity of ROP signalling

**Fig. 3.**
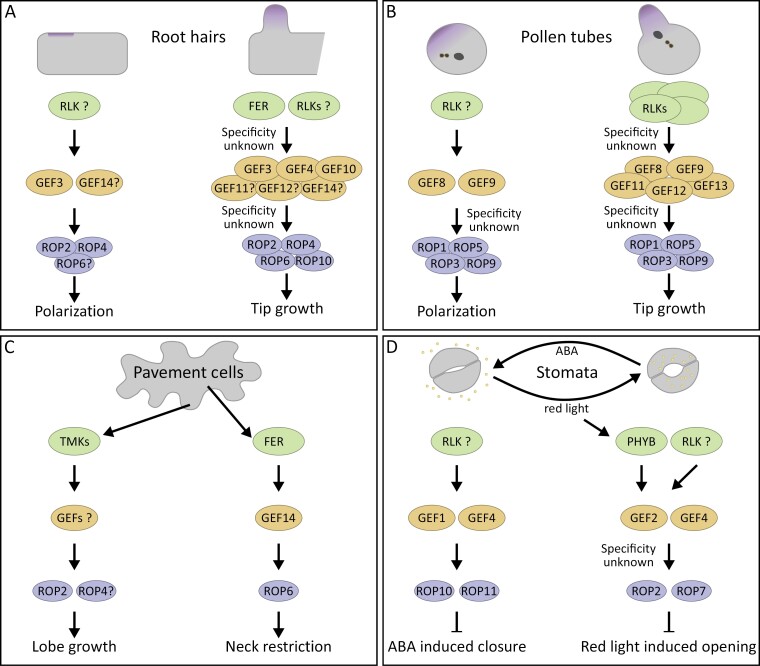
RHO OF PLANTS (ROP) GUANINE NUCLEOTIDE EXCHANGE FACTORS (GEFs) drive the specificity of ROP signalling. Different examples in which ROPGEFs specifically activate one process but not others. The participating ROPGEFs are present simultaneously in all the cases shown, but only their specific downstream process is activated. This might depend on spatiotemporal differences or different binding properties from other components of different ROP signalling complexes. (A) Root hair initiation and tip growth are regulated by overlapping ROPs but are activated by different ROPGEFs. Polarization of ROP signalling at the root hair initiation domain is driven by ROPGEF3 and potentially ROPGEF14. Their activating receptor remains unknown, and only ROP2 and ROP4 were shown to regulate root hair initiation specifically, but it is likely that ROP6 also participates in this process. Multiple ROPGEFs regulate root hair tip growth, but a function is shown only for ROPGEF3, ROPGEF4, and ROPGEF10. The other shown ROPGEFs are expressed in root hairs, but no function is established yet. The RECEPTOR-LIKE KINASE (RLK) FERONIA (FER) regulates GEF4 and GEF10, while potential other RLKs are needed to activate all expressed GEFs. So far, no specificity of ROPGEFs to ROPs has been determined, which could help us understand the individual ROP-regulated pathways. (B) Pollen germination and pollen tube tip growth are regulated by the same ROPs but are activated by different ROPGEFs. ROPGEF8 and ROPGEF9 specifically accumulate at the pollen germination site and polarize ROP signalling. Their activating RLK still needs to be determined. During pollen tube growth, multiple RLKs are known to activate various ROPGEFs, activating the same set of ROPs. It remains to be determined if any specificities of RLKs towards ROPGEFs or ROPGEFs towards ROPs occur in activating downstream pathways. (C) The TRANSMEMBRANE KINASE (TMK) RLKs activate ROP2 and potentially ROP4 via an unknown ROPGEF to drive auxin-dependent lobe growth in pavement cells. On the other hand, FER activates ROP6 via GEF14 to restrict growth in the neck regions. It still needs to be determined which ROPGEF mediates TMK signalling, if another ROPGEF is also present in the FER-dependent pathway, and how specificity between both pathways is achieved. (D) ROPGEF1 and ROPGEF4 inhibit ABA-induced stomata closure via ROP10 and ROP10. However, their activation mechanism has yet to be discovered. On the other hand, ROPGEF2 and ROPGEF4, together with ROP2 and ROP7, inhibit PHYTOCHROME B (PHYB)-dependent stomatal opening in response to red light. PHYB directly activates ROPGEF2 to counteract its function, but it still needs to be revealed how ROPGEF4 is activated. The specificities of these co-occurring pathways still need to be determined, especially for ROPGEF4, which is part of both processes with opposite results.

In most cells, multiple ROPGEFs are present and are thought to act redundantly. In most studied processes, higher-order mutants were required to observe phenotypes. ROPGEF4 and ROPGEF10 act together during root hair growth. At the same time, ROPGEF1 and ROPGEF4 regulate abscisic acid (ABA) signalling, or ROPGEF8, ROPGEF9, ROPGEF11, ROPGEF12, and ROPGEF13 control cell integrity during pollen tube growth (Li and Liu, 2012; Huang *et al.*, 2013; Zhou *et al.*, 2023). This requirement of multiple ROPGEFs for one cellular process is similar to the described redundancy of ROPs (Li *et al*., 1998; Xiang *et al.*, 2023). It implies no specificities or functional differences between the individual members of the protein family. However, as was shown for RIC3 and RIC4 in pollen tube growth and discussed earlier in this review, ROP signalling complexes can specifically activate different cellular pathways, depending on the complex composition (Gu *et al.*, 2005). This does argue against redundant molecular functions of individual components of the ROP signalling pathway. In line with this, we demonstrated in root hair cells that individual ROPGEFs can regulate specific aspects of polar growth within the same cell (Fig. 3). Only ROPGEF3 polarizes ROPs to the root hair initiation site during root hair initiation, while ROPGEF4 does not affect this process (Denninger *et al.*, 2019). ROPGEF4 activates ROPs only later in tip-growing root hairs together with ROPGEF10 (Huang *et al.*, 2013). This proves that ROPGEFs do not act redundantly in some processes and indicates that individual ROPGEFs have distinct functions to activate specific aspects of ROP signalling. Similar specificities are present during pollen germination, where ROPGEF8 and ROPGEF9 are crucial for ROP signal polarization, while ROPGEF12 and ROPGEF11 do not show such polarization (Bouatta *et al.*, 2024, Preprint). In pollen tube growth, the C-terminus of ROPGEFs is essential for the interactions with PRKs and subsequent activation of ROP signalling (Zhang and McCormick, 2007; Chang *et al.*, 2013). ROPGEF8 and ROPGEF12 show the same interaction specificity towards PRKs (Takeuchi and Higashiyama, 2016; Yu *et al.*, 2018). However, the polarization of ROPGEFs in pollen is specific to the C-terminus of ROPGEF8 and ROPGE9 but not GEF12 (Bouatta *et al.*, 2024, Preprint). This indicates an unknown, GEF8/9-specific mechanism that polarizes these ROPGEFs in pollen germination and further demonstrates that ROPGEFs do not act redundantly on a molecular level (Fig. 3). Whether these specificities are only relevant for initiating cell polarization or if such specificities are also found in other aspects of ROP signalling still needs to be investigated. ROPGEFs may define the output of ROP signalling by controlling the complex composition from the beginning of its assembly to ensure the specific functions of the signalling pathway.

## Cellular and developmental processes regulated by ROPGEFs

All processes that involve polar growth or reaction to a local signal require cell polarity and underlying mechanisms like local cell wall loosening, polar exocytosis, or cytoskeletal rearrangement (Rounds and Bezanilla, 2013). All these mechanisms have been linked to ROP signalling in plants, but in many cases, our knowledge about all required regulators is still incomplete (Feiguelman *et al.*, 2018). Here, I will discuss known ROP-regulated developmental and cellular processes for which the role of ROPGEF proteins is known and highlight the processes for which the function of ROPGEFs still needs to be described.

### Tip growth

Tip growth is the most studied model for ROP signalling, and many connections to cellular mechanisms have been described. ROP signalling regulates root hair initiation and pollen germination, tip growth in these systems, and other tip-growing cells like the protonema of *Physcomitrium patens* (Li *et al*., 1999; Rounds and Bezanilla, 2013; Bascom *et al.*, 2019). ROPGEF3 initiates polarization in root hairs, while ROPGEF8 and ROPGEF9 have this function in pollen germination (Denninger *et al.*, 2019; Bouatta *et al.*, 2024, Preprint). During tip growth, more ROPGEFs are required (Fig. 3). ROPGEF3, ROPGEF4, and ROPGEF6 of *Physcomitrium patens* have been visualized in the tip of the growing protonema cell (Le Bail *et al.*, 2019; Yoshida *et al.*, 2023). ROPGEF4 and ROPGEF10 were shown to link FER signalling to ROP signalling in growing root hairs (Huang *et al.*, 2013). During pollen tube growth, ROPGEF8, ROPGEF9, ROPGEF11, ROPGEF12, and ROPGEF13 are crucial for normal growth and to maintain cellular integrity in changing cellular constrictions while the pollen is growing in the transmitting tract of the flower (Zhou *et al.*, 2023). ROP signalling regulates many cellular processes in these cells. Still, we need to know the specificities of ROPGEFs towards these individual processes or which proteins are required to connect these pathways. For example, the exocyst complex component Sec3a, which links the exocyst complex to the plasma membrane during exocytosis, is required for root hair and pollen growth (Wen *et al.*, 2005; Zhang *et al*., 2013; Bloch *et al*., 2016; Žárský, 2022). Moreover, Sec3a polarizes at the pollen germination site, similar to ROPGEF8 and ROPGEF9 (Bloch *et al*., 2016; Li *et al*., 2017; Bouatta *et al.*, 2024, Preprint). An interaction between Sec3a and the ROP effector RIP1 was shown (Lavy *et al.*, 2007). However, it is still to be determined if this interaction solely depends on downstream scaffold proteins or if early factors like ROPGEFs or ROPs also interact directly with exocyst components (Žárský, 2022). Additionally, more cell types, like papilla cells or the elongating zygote, have tip-growing mechanisms (Kimata *et al.*, 2016). However, the ROPGEFs and other ROP signalling components involved in these processes remain elusive. Studying the different ROP signalling components in these additional systems will help in understanding the general role of ROP signalling in tip-growing cells.

### Pollen perception and cell–cell recognition

On the male side of plant fertilization, we have a broad knowledge of ROP signalling and know which ROPs and ROPGEFs are required for pollen activation and germination after they come in contact with the floral tissue. However, we need to learn more about the ROP signalling involved in papilla cells, which perceive the pollen grains. Multiple RLKs were shown to be involved in the cellular responses towards compatible and incompatible pollen. For example, the RKLs ANJEA and FER activate high ROS production by NOX/RBOHs in papilla cells to generate conditions that prevent alien pollen or pathogens from growing. Compatible pollen inhibits the FER/ANJ-dependent ROS production, allowing pollen germination and growth. The FER/ANJ-dependent ROS production is mediated by ROP2, but no other factors are described (Liu *et al*., 2021; Zhang *et al*., 2021). It remains to be shown which ROPGEFs and scaffold proteins are essential in this process and if other ROPs mediate different aspects of pollen recognition in papilla cells, as many other RLK signalling pathways are required for this cell–cell recognition. These includes RECEPTOR-LIKE KINASE IN FLOWERS (RKF) that generally activate pollen hydration or S-LOCUS RECEPTOR KINASEs (SRK) that recognize self-incompatible pollen in *Brassica* and prevent its growth (Takayama *et al.*, 2001; Lee and Goring, 2021). In both cases, it needs to be determined if ROPGEFs or other ROP signalling components are involved. This also applies to other cell–cell recognition processes during fertilization. The synergid cell FER–ANJ–HERK1 receptor complex recognizes the pollen tube and triggers pollen tube burst and synergid cell death (Escobar-Restrepo *et al.*, 2007; Galindo-Trigo *et al.*, 2020). However, the downstream pathways of these RLKs have yet to be studied, and it remains to be investigated if the same ROP signalling proteins, like in other FER-dependent processes, are required.

## Immune responses

Similar to the perception of other cells during fertilization, host immune responses recognize alien cells and must decide if they are from a pathogen or a useful symbiont. The reaction of the host cell is a highly polar process, and in different species ROPs are involved in the recognition process of alien organisms (Engelhardt *et al.*, 2020). In Arabidopsis, ROP6 functions in immune responses against fungal pathogens and, together with ROP1, in symbiotic interactions. ROP signalling affects ROS production and actin rearrangements in both cases (Poraty-Gavra *et al.*, 2013; Venus and Oelmüller, 2013). However, how the specificity towards a pathogen or symbiont is achieved when the same ROP regulates both processes is still being determined. In Arabidopsis, ROPGEFs that might be involved in early polarization processes during biotic interactions still need to be investigated. Similarly, ROPs were shown to be required for nodulation in legumes, but the ROPGEFs still need to be identified (Ke *et al.*, 2012). Compared with the model plant Arabidopsis, ROP signalling has been described in more detail in crop species. The rice ROP RAC1 enhances NOX/RBOH-dependent ROS production during pathogen responses and is activated by RACGEF1, which is directly associated with the pathogen receptor (Nakashima *et al.*, 2008; Akamatsu *et al.*, 2013). In barley, multiple ROPs modulate defence responses during fungal invasions. RIC and RIP scaffold proteins are connected to these ROP signalling pathways to activate polar cytoskeleton rearrangements towards the invasion site (Pathuri *et al.*, 2008; Hoefle *et al.*, 2020; Engelhardt *et al.*, 2023). Furthermore, barley ROPGEF14 was identified to activate ROPs during pathogen responses (Trutzenberg *et al.*, 2022). Despite our knowledge of multiple ROP signalling components during biotic interactions, considering the significant number of potential interactions of plant cells with other organisms, which all require specific cellular responses, our knowledge still needs to be improved to understand how plants generally react to other organisms and how they can discriminate between friend and foe.

### Pavement cell growth

The described ROP-dependent pathways all regulated processes in individual specialized cells. However, ROP signalling also affects general plant growth and organ development. An established model for the role of ROP signalling in cellular growth and organ development is the interdigitation of leaf epidermis pavement cells (Fu *et al*., 2005; Feiguelman *et al.*, 2018). In this growth process, a cell forms a lobe by polarly growing cells in a defined region, causing a neck structure in its neighbouring cells (Fig. 3). A ROP-dependent negative feedback loop confines this growth to a small region, allowing the neighbouring cell to form a lobe adjacent to its neck (Fu *et al*., 2005; Xu *et al.*, 2010; Chen *et al*., 2015).  This alternating polar growth leads to puzzle-shaped pavement cells. To constrict growth in the neck region, the cell wall pectins are demethylated and thus locally harden the cell. The RLK FER binds these cell wall structures and activates ROP6 via ROPGEF14. ROP6 activates microtubule polymerization and confinement, which prevents growth in the neck region. In contrast, ROP2 drives lobe growth by activating actin polymerization (Xu *et al.*, 2010; Lin *et al*., 2022). An additional, brassinosteroid-dependent pathway specially regulates ROP2. BIN2 phosphorylates and stabilizes PHGAPs in neck regions, specifically inactivating ROP2 but not ROP6. How this specificity is achieved still needs to be determined. In the lobe region of the opposite cell, the brassinosteroid receptor BRASSINOSTEROID INSENSITIVE 1 (BRI1) inactivates BIN2, which causes PHGAP degradation and releases ROP2 inhibition (Lauster *et al.*, 2022; Zhang *et al*., 2022). Additionally to this brassinosteroid-dependent pathway, a second pathway that depends on the phytohormone auxin activates ROP2 in lobe regions (Fig. 3). Extracellular auxin is sensed by TRANSMEMBRANE KINASE (TMK) RLKs, which activate ROP2 and thus actin-dependent polar growth (Xu *et al*., 2014; Yu *et al.*, 2023). The ROPGEF activating ROP2 still needs to be identified.  This is also the case for the connection to the sole DOCK-GEF SPIKE1, which is proposed to activate ROP6 in pavement cells (Basu *et al.*, 2008). These opposing pathways, in which two different ROP signalling pathways counteract each other, are a great example of the importance of understanding the individual ROP complexes and their downstream pathways. They can serve as a blueprint for many other ROP-dependent processes (Fig. 3).

## Polar cell growth in plant organ development

Despite pavement cell interdigitation, ROP-activating TMK auxin receptors control many developmental processes in which cells polarly elongate. This indicates that ROPs are involved in all these auxin-dependent growth processes. The severity of lacking ROP signalling was shown in different ROP signalling mutants. Mutations in the genes for ROP3, RIP1/ICR1, ROPGEF1, and ROPGEF7 cause general developmental defects during embryo development and in seedlings (Lavy *et al.*, 2007; Hazak *et al.*, 2010; Chen *et al.*, 2011; Huang *et al.*, 2014; Liu *et al.*, 2017). These range from altered cell division patterns to reduced cell elongation. All these ROP-signalling components were linked to auxin signalling and distribution. ROP3 and ICR1 were proposed to increase the polarity of PINFORMED (PIN) auxin exporters (Hazak *et al.*, 2010; Huang *et al.*, 2014). ROPGEF1 was shown to regulate the correct polarization of the auxin influx carrier AUX1 in embryo development and PIN2 in root meristems, which causes embryonic defects or loss of gravitropic responses, respectively (Liu *et al.*, 2017). Even though ROP signalling might be involved in most developmental processes and translates different hormone signals into polar cellular responses, our knowledge about the involved ROP-signalling components is limited.

### Stomatal closure

The aperture of stomata is regulated by light and water availability to ensure optimal water transport and photosynthesis. Signalling by the phytohormone ABA acts in a negative feedback loop with ROP signalling to regulate stomatal closure (Fig. 3). ABA leads to the degradation of ROP signalling components. At the same time, ROPGEF1 and ROPGEF4, together with ROP11 and ROP10, were shown to inhibit ABA-dependent stomata closure (Li and Liu, 2012). The photoreceptor PHYTOCHROME B (PHYB) mediates stomatal opening in response to red light. Additionally, PHYB activates ROPGEF2 but not ROPGEF4 and, subsequently, ROP2 and ROP7 to suppress PHYB-dependent stomatal opening and to prevent excessive stomatal opening (Fig. 3) (Wang *et al.*, 2017). Even though many components of the ROP signalling pathway are described, the activators of ROPGEF1 and ROPGEF4 still need to be determined for both responses. Moreover, it remains to be seen how specificity in these processes is achieved and how crosstalk is prevented. This is especially interesting for ROPGEF4, which positively regulates stomatal opening in ABA signalling and negatively regulates stomatal opening in response to red light (Fig. 3).

## Cellular differentiation and subcellular polar domains

ROP signalling is connected to many polar processes, but ROPs additionally regulate cell differentiation processes that require defined polar domains. In xylem differentiation, the xylem vessel’s cell wall is reinforced by a lignified secondary wall. To allow for lateral water movements and connections to the surrounding tissue while keeping a structure resilient against water pressure, xylem cell walls are interrupted by cell wall pits in metaxylem cells. These cell wall pits are round structures of similar size of a few micrometres diameter, regularly spaced over the whole surface of metaxylem vessels (Růžička *et al.*, 2015; Xu *et al*., 2022). ROP signalling is crucial for establishing these cell wall pits by local induction of microtubule depolymerization. ROPGEF4 and ROPGEF7, in combination with ROPGAP3 and ROPGAP4, regulate ROP11 activity and the downstream scaffold proteins RIP3/ICR5/MIDD1 and BDR1 (Oda and Fukuda, 2012; Sugiyama *et al.*, 2017, 2019; Nagashima *et al.*, 2018; Feiguelman *et al.*, 2022). RIP3 interacts with KINESIN13A, which has microtubule destabilizing properties. BDR1 promotes actin polymerization at the pit edges to confine ROP activity to the pit region (Sugiyama *et al*., 2019). Establishing these polar membrane domains is based on the intrinsic clustering properties of the ROP complex following a Turing-style reaction–diffusion mechanism (Jacobs *et al.*, 2020; Deinum and Jacobs, 2024). Depending on the composition of such complexes, different domain patterns can occur and recruit downstream factors. The formation of xylem pits shows that the composition of ROP complexes is crucial and that ROPGEF and ROPGAPs are required simultaneously in these ROP complexes to form stable signalling domains and achieve a balanced ROP activity by a fine-tuned negative feedback loop (Xu *et al*., 2022; Deinum and Jacobs, 2024). In contrast to the ROP-dependent formation of cell wall patterns in metaxylem cells, little is known about the formation of spiral cell wall patterns in protoxylem cells. A RIP3/MIDD1 diffusion restriction mechanism by microtubules together with ROP7, ROP8, and ROP11 regulates this patterning, and mathematical modelling supports this mechanism, but experimental data on other ROP signalling components are missing (Jacobs *et al.*, 2020; Higa *et al.*, 2024). Furthermore, ROP signalling might regulate similar locally defined cell wall patterns, such as in the casparian strip, the chalazal region of the egg cell, or in sieve plate formation in phloem cells (Geldner, 2013; Tekleyohans *et al.*, 2017; Kalmbach and Helariutta, 2019). No ROP signalling components have been associated with these processes, but ROP9, ROPGEF2, ROPGEF3, and ROPGEF5 are up-regulated in developing phloem cells and are enriched at the apical–basal cell sides, which suggests a function of these proteins in phloem cell wall patterning (Roszak *et al.*, 2021).

## Cell division

Similar to the formation of xylem pits, in cell division a specific, restricted region in the cell must be defined to determine cell division orientation. In plant cells, the orientation of the division plane is one of the first steps of mitosis. In pre-prophase, a band of microtubules and actin surrounds the cell and defines the cell division plane. While this pre-prophase band does not persist during mitosis, some of its components permanently localize to this cortical division zone and guide the newly formed cell plate to this location (Müller *et al.*, 2009; Lipka *et al.*, 2015; Yi and Goshima, 2022). In asymmetric cell division of subsidiary cells of monocotyledon stomata, actin and ROP signalling are in a positive feedback loop to define cellular asymmetry. This process depends on the grass-specific PANGLOSS RLKs, which activate ROPs on one side of the cell (Humphries *et al.*, 2011; Facette *et al.*, 2015; Yi and Goshima, 2022). The pre-prophase band and cortical division zone are adjacent to this region. Thus, ROP signalling defines the cell division plane. However, the required ROPGEFs for this process still need to be determined. Furthermore, it still needs to be determined if similar RLKs act during cell division of other plant species and how the orientation of cell division is initially selected. Membrane-associated proteins like BREVIS RADIX (BRX), POLAR, and OCTOPUS-LIKE (OPL) can determine cell orientation and membrane domains, leading to a defined location of the cell division plane and pre-prophase band formation (Wallner *et al.*, 2023). In Arabidopsis, the pre-prophase band is initiated by a combination of gradual actin and microtubule reorientation and enrichment, directed by the multi-protein TTP complex, consisting of TONNEAU1 (TON1), TON1 RECRUITMENT MOTIF (TRM) proteins, and PROTEIN PHOSPHATASE2A (PP2A). It still needs to be determined if ROP signalling is required for this initial cell division site determination, similar to subsidiary cell division. However, the importance of cytoskeleton rearrangement in a defined region at the plasma membrane indicates the role of ROP signalling in this process (Lipka *et al.*, 2015; Yi and Goshima, 2022). After establishing the pre-prophase band, PHGAP proteins accumulate at this site and persist throughout cell division (Stöckle *et al.*, 2016; Yi and Goshima, 2020). These negative ROP regulators are required for maintaining the cortical division zone orientation and microtubule-dependent cell plate guidance during cytokinesis. PHGAPs interact with the microtubule-associated PHRAGMOPLAST ORIENTING KINESIN (POK) and IQ67 DOMAIN (IQD) proteins, but the mechanism of PHGAP function and which ROP proteins they regulate still need to be determined (Kumari *et al.*, 2021). Interestingly, ROPGEF and ROP activity seem to be excluded from the PHGAP-defined cortical division zone in developing phloem cells, suggesting negative feedback on ROP accumulation and signalling by PHGAPs at the cortical division site (Roszak *et al.*, 2021). That ROP signalling has a general role in cell division can be seen in multiple organisms. Multiple RIP proteins in Arabidopsis, or the ROP of *Marchantia polymorpha*, are required for cell division patterning and cell division plane orientation (Hasi and Kakimoto, 2022; Rong *et al.*, 2022; Mulvey and Dolan, 2023b). In *Physcomitrium patens*, ROPs orient cell division towards a branching protonema cell, which is determined early by ROPGEF4 (Yi and Goshima, 2020; Ruan *et al.*, 2023). However, it still needs to be determined which cell division phases and processes are regulated by ROP signalling and whether this depends on a single ROP complex or distinct ROP complexes. Furthermore, the remaining components of the ROP signalling complex still need to be determined in all cases. Nevertheless, multiple lines of data support the idea that ROP signalling has a fundamental cell biological function, which should be investigated to understand plant cell division and development.

## Conclusion

ROP signalling regulates a plethora of processes in plants, and we constantly gain knowledge about the individual components of these signalling pathways. However, the cellular output of ROP signalling and the regulated cellular processes can vary depending on the ROP complex composition. Multiple examples demonstrated that such different ROP complexes can co-occur within one cell. Different ROPGEFs have distinct functions within one cell and regulate various cellular processes. Thus, it is insufficient to describe one aspect or component of these pathways to understand the cellular function. It is crucial to determine all ROP signalling components within a cell, link them with each other, and investigate their spatiotemporal connection. This holistic understanding of ROP signalling will be required to understand all aspects of ROP signalling and its role in cellular growth, cell polarity, cell differentiation, and overall plant development.
